# Bioactive Composite Cryogels Based on Poly (Vinyl Alcohol) and a Polymacrolactone as Tissue Engineering Scaffolds: In Vitro and In Vivo Studies

**DOI:** 10.3390/pharmaceutics15122730

**Published:** 2023-12-05

**Authors:** Bianca-Elena-Beatrice Crețu, Gianina Dodi, Ioannis Gardikiotis, Vera Balan, Isabella Nacu, Iuliana Stoica, Elena Stoleru, Alina Gabriela Rusu, Alina Ghilan, Loredana Elena Nita, Aurica P. Chiriac

**Affiliations:** 1 Department of Natural Polymers, Bioactive and Biocompatible Materials, Petru Poni Institute of Macromolecular Chemistry, 41 A Grigore Ghica Voda Alley, 700487 Iasi, Romania; cretu.bianca@icmpp.ro (B.-E.-B.C.); cobzariu.isabella@gmail.com (I.N.); rusu.alina@icmpp.ro (A.G.R.); diaconu.alina@icmpp.ro (A.G.); achiriac@icmpp.ro (A.P.C.); 2 Biomedical Sciences Department, Faculty of Medical Bioengineering, Grigore T. Popa University of Medicine and Pharmacy of Iasi, 9-13 Kogalniceanu Street, 700454 Iasi, Romania; gianina.dodi@umfiasi.ro (G.D.); balan.vera@umfiasi.ro (V.B.); 3 Advanced Research and Development Center for Experimental Medicine, Grigore T. Popa University of Medicine and Pharmacy of Iasi, 9-13 Kogalniceanu Street, 700454 Iasi, Romania; ioannis.gardikiotis@umfiasi.ro; 4 Department of Physical Chemistry of Polymers, Petru Poni Institute of Macromolecular Chemistry, 41 A Grigore Ghica Voda Alley, 700487 Iasi, Romania; stoica_iuliana@icmpp.ro (I.S.); elena.paslaru@icmpp.ro (E.S.)

**Keywords:** poly(vinyl alcohol), copolymacrolactone, thymol, α-tocopherol, cryogel, wound healing

## Abstract

In light of the increasing resistance of pathogenic microorganisms to the action of antibiotics, essential oils extracted from plants with therapeutic activity provide a significant alternative to obtaining dressings for the treatment of skin wounds. The encapsulation of essential oils in an amphiphilic gel network allows better dispersion and preservation of hydrophobic bioactive substances while promoting their prolonged release. In this study, we focused on the development of a poly (vinyl alcohol) (PVA)/poly (ethylene brassylate-co-squaric acid) (PEBSA) platform embedded with thymol (Thy), and α-tocopherol (α-Tcp) as a co-drug structure with prospective use for the treatment and healing of skin wounds. The new complex bioactive system was prepared through repeated freeze–thaw processes. The influence of the composition on surface topography, hydrophilic/hydrophobic character, and in vitro interaction with simulated body fluids was evidenced. BALB/3T3 fibroblast cell culture demonstrated the cryogel scaffolds’ cytocompatibility. Tests on Wistar rats confirmed their biocompatibility, integration with host tissue, and the absence of inflammatory processes. The bioactive compound significantly enhanced the healing process of full-thickness excision wounds in a rat model. Further investigations on in vivo infection models would assess the potential of the PVA/PEBSA platform with dual bioactive activity for clinical antimicrobial and wound healing therapy.

## 1. Introduction

Bacterial infections are a common issue associated with dermal wounds [1], contributing to a slow healing process and even necrosis of local tissues [2]. The main goal of wound management is to enhance the healing of wounds within the shortest possible time by preventing infections and minimizing pain, inflammation, and discomfort [3]. Materials such as cotton and gauze are widely used as wound dressings because they insulate the wound from the outside environment, but they do not exhibit antibacterial properties and are not suitable for all types of wounds. In addition, wound closure rates are almost twice as high in wet conditions as in dry conditions, hence dry cotton and gauze dressings are gradually being replaced by wet dressings [4]. Hydrogels are considered the ideal substitute for dry wound dressings due to their high water content, biocompatibility, and structural similarity to natural biological tissues [5]. Hydrogel dressings can absorb and maintain an optimal level of exudate on the wound surface, allowing oxygen to reach the wound for cell proliferation [6]. Hydrogel transparency is an additional advantage over traditional materials because wound healing can be constantly monitored without removing the dressing. Despite these properties, conventional hydrogels face challenges in their widespread implementation due to their closed and small cavities. Cryogels exhibit superior characteristics, given their large pore size, increased elasticity, and heightened strength attributed to the presence of crystalline regions [7,8,9]. PVA is one of the most investigated water-soluble synthetic polymers for obtaining cryogels, with applications as a drug carrier, in wound dressings, and for tissue engineering due to its biocompatibility, biodegradability, and non-toxicity [10,11]. The use of pure PVA-based cryogels as a biomaterial is limited by the absence of bioactivity and inertia in the healing process of skin wounds [12,13]. To overcome these limitations, combining them with other materials or compounds with antimicrobial properties and/or stimulatory effects on growth factors is a promising approach for wound healing [14]. Foroughi et al. [15] designed a PVA/chitosan/gum tragacanth hydrogel loaded with vitamin E through the repeated freezing–thawing approach for wound healing applications. The results indicated that materials containing chitosan and tragacanth exhibited the highest antimicrobial activity against *Staphylococcus aureus* (*S. aureus*—11 mm of inhibition zone) and *Escherichia coli* (*E. coli*—8 mm of inhibition zone), a good release rate, acceptable cytocompatibility, and cellular adhesion. Xiong et al. [4] used PVA, chitosan, and vanillin to prepare a three-phase hydrogel dressing using the freeze–thaw technique. Their evaluation tests showed excellent mechanical and biological properties, with potent antioxidant properties (>70%) and the ability to inhibit the growth of *E. coli* (~6 mm) and *S. aureus* (~5 mm).

In a previous publication [16], our group employed this approach to create novel cryogels with antimicrobial and antioxidant properties. These cryogels were based on PVA, PEBSA, Thy, and α-Tcp, synthesized through a repeated freeze–thaw process. Thy exhibited antimicrobial activity against *Candida albicans* (*C. albicans*—up to 38 mm of inhibition zone)*, S. aureus* (>22 mm of inhibition zone), and *E. coli* (>21 mm of inhibition zone), while the encapsulation of α-Tcp conferred antioxidant properties (93.2–97.1%) to the PVA/PEBSA_Thy system. This combination aimed to achieve a dual therapeutic effect by incorporating both bioactive molecular agents. Taking into account the previous results, the present study is dedicated to the potential applicability as a wound dressing of this new material. Thus, the influence of cryogels’ preparation conditions on surface topography, hydrophilic/hydrophobic character, and in vitro interaction with simulated body fluids was studied. The applicability of the new complex bioactive system was investigated through cell proliferation, a biocompatibility test, and an in vivo wound healing assay in a full-thickness excision wound rat model. 

## 2. Materials and Methods

### 2.1. Materials

Ethylene brassylate (EB, 1,4-dioxacycloheptadecane-5,17-dione, C_15_H_26_O_4_, M_w_ = 270.36 g/mol, purity of 95.0%), squaric acid (SA, 3,4-dihydroxy-3-cyclobutene-1,2-dione, H_2_C_4_O_4_, M_w_ = 114.06 g/mol, purity > 99.0%), (+)-α-Tocopherol (α-Tcp), calcium chloride dihydrate (CaCl_2_·2H_2_O), magnesium chloride hexahydrate (MgCl_2_·6H_2_O), potassium chloride (KCl), sodium chloride (NaCl), sodium sulfate (Na_2_SO_4_), monosodium phosphate (Na_2_HPO_4_·2H_2_O), disodium phosphate (Na_2_H_2_PO_4_·7H_2_O), sodium hydrogen carbonate (NaHCO_3_), tris-hydroxymethyl-aminomethane ((CH_2_OH)_3_CNH_2_), hydrochloric acid (HCl), and 1,4-dioxane (purity ≥ 99.0%) were purchased from Sigma-Aldrich (Steinheim, Germany); poly(vinyl alcohol) (PVA, M_w_ = 72,000 g/mol and M_w_ = 145,000 g/mol, 98% hydrolyzed) was acquired from Merck (Hohenbrunn, Germany); thymol (Thy, 2-isopropyl-5-methylphenol, C_10_H_14_O) was obtained from Alfa Aesar (Kandel, Germany); and anhydrous 1-hexanol (H_3_C(CH_2_)_4_CH_2_OH) was obtained from Acros Organics (Geel, Belgium). All reagents were analytical grade and used as received.

### 2.2. Preparation of Cryogels

The cryogels were prepared through the repeated freeze–thaw technique, as mentioned in the previous publication of our group [17], briefly described below. PEBSA, the polymacrolactone synthesized by polycondensation of EB macrolactone through ring-opening with SA in different molar ratios [18], was mixed with Thy and α-Tcp in 1,4-dioxane (1:1:1 ratio *w*/*w*/*w*) to form the bioactive complex [16]. Subsequently, the obtained complex was combined with PVA solution (4%, *w*/*v*) in a volumetric ratio of 2:1, followed by repeated cycles of freeze-drying at −20 °C for 18 h and thawing at 25 °C (ambient temperature) for 8 h. The prepared cryogels were frozen with liquid nitrogen and lyophilized for 24 h at −55 °C (Alpha 1-2LD Plus, Martin Christ, Germany). The systems were codified, according to their composition, as detailed in Table 1.

### 2.3. Characterization

Both PVA/PEBSA_Thy_α-Tcp bioactive compounds and the PVA/PEBSA matrix were previously characterized from a physicochemical point of view, namely, their chemical composition, thermal stability, network morphology, release profiles, as well as their antimicrobial and antioxidant properties [13,16,17]. In the present study, our group evaluates first the influence of cryogels’ preparation conditions on the surface topography and hydrophilic/hydrophobic character, followed by in vitro and in vivo studies.

The in vivo experiments followed the 3R principles aimed at reducing the number of animal experiments; therefore, due to a limited number of animals according to the Materials and Methods section, a selection of analyzed samples was made. The superior antibacterial antimicrobial activity against *C. albicans* (up to 38 mm of inhibition zone) and antioxidant properties (97.1%) [16], moderate hydrophilic character (~55°), and significant swelling capacity (871%) favored the selection of the bioactive compound PVA_72000__PEBSA_25/75__Thy_α-Tcp as the experimental group. The PVA_72000__PEBSA_25/75_ system was selected as the positive control.

#### 2.3.1. Atomic Force Microscopy (AFM)

The surface topographical features of the obtained cryogels based on PVA and PEBSA copolymacrolactone were investigated by AFM, in atmospheric conditions, at room temperature using an NTEGRA Scanning Probe Microscope from NT-MDT Spectrum Instruments (Moscow, Russia). The resonance frequency of the NSG10 cantilever utilized in tapping mode was 241 kHz. Nova 1.0.26.1443 and Image Analysis 3.5.0.19892 software were employed for data acquisition and to calculate the 3D roughness parameters on the 10 × 10 µm^2^ selected images.

#### 2.3.2. Water Contact Angle (WCA)

The static WCA was determined by the sessile drop method using a CAM-200 from KSV Instruments (Helsinki, Finland), at room temperature and ~55% relative humidity, within 1 s after placing a 1 μL drop of water on the polymeric film surface. WCA measurements were made at least 5 times, on different surface areas, and average values were reported.

#### 2.3.3. In Vitro Interaction with Simulated Body Fluids

Swelling degree in simulated body fluid (SBF). The swelling behavior of freeze-dried cryogels was studied in SBF solution at 37 °C using the gravimetric method. SBF solution was prepared as described in [19] by dissolving reagent-grade NaCl, NaHCO_3_, KCl, Na_2_HPO_4_·2H_2_O, MgCl_2_·6H_2_O, CaCl_2_·2H_2_O, and Na_2_SO_4_ into ultrapure water, adjusted to pH 7.4 and 37 °C with (CH_2_OH)_3_CNH_2_) and 1 M HCl solution. At defined intervals of time, the weight of the samples was noted after collecting them from the SBF solution and blotting the surface liquid excess with filter paper. The swelling degree was calculated by using Equation (1):

(1) $$Swelling\;degree,\%=\frac{W_{t}-W_{0}}{W_{0}}\times 100$$

where W_t_ is the weight of the swollen cryogels at a particular time t and W_0_ is the initial weight of the dry samples (g).

Equilibrium swelling degree in sodium phosphate buffer (PBS). The pH sensitivity of the samples was also evaluated in PBS solution at different pH values (5.4, 5.6, 6, 6.4, 7, 7.4, and 8, 0.01 M) using the same method. Each cryogel sample was weighed and immersed in PBS solutions until equilibrium swelling was reached. The equilibrium swelling degree was calculated by using Equation (2):

(2) $$Equilibrium\;swelling\;degree,\%=\frac{W_{\infty }-W_{0}}{W_{0}}\times 100$$

where W_∞_ is the weight of the cryogels in the swollen state at equilibrium and W_0_ is the initial weight of dry samples (g).

#### 2.3.4. In Vitro Cytocompatibility

##### MTT Assay

All reagents/kits used for cell culture were purchased from Sigma-Aldrich (Steinheim, Germany) unless otherwise specified. Mouse fibroblasts (cell line BALB/3T3 clone A31) were cultivated in Dulbecco’s Modified Eagles Medium—high glucose (DMEM, Biowest, France) supplemented with 10% fetal bovine serum and 1% penicillin/streptomycin/neomycin mixture (~5000 units penicillin, 5 mg streptomycin, and 10 mg neomycin/mL) under standard cell culture conditions (95% relative humidity, 5% CO_2_, and 37 °C). The cells were seeded in a 48-well plate (15 × 10^3^ cells/100 µL, with 400 µL of complete DMEM) and maintained for 24 h in a humidified atmosphere, 5% CO_2_ at 37 °C. The selected scaffolds were sterilized by exposure to UV light (30 min on each side) followed by addition on the cell layers and incubation for 72 h.

To perform the MTT assay, the medium was replaced with Thiazolyl Blue Tetrazolium Bromide working solution (MTT, 5% in simple DMEM) and incubated at 37 °C for 3 h, protected from light. Formazan was solubilized with dimethyl sulfoxide, 500 μL/well, and the absorbance was read at 570 nm (on Tecan Sunrise Plate Reader, Switzerland). The percentage of cell viability was calculated over time (at 24, 48, and 72 h) using Equation (3):

(3) $$Cell\;viability,\%=\frac{{Abs}_{sample}}{{Abs}_{control}}\times 100$$

where ${Abs}_{sample}$ is the absorbance of the cells with scaffold and ${Abs}_{control}$ is the absorbance of the control cells. In order to sustain MTT results, cell morphology and density were studied, using an Inverted Phase Contrast Microscope (Leica, Wetzlar, Germany) and images were taken at 10× lens magnification. All measurements were performed in triplicate.

##### Live/Dead Cell Staining

A live/dead-staining assay with Calcein-AM was used to investigate the distribution of cells on the obtained scaffolds after 72 h. The dye solution (1 μM Calcein-AM/mL in Hanks’ Balanced Salt Solution) was incubated in cell cultures at 37 °C for 30 min, followed by cell imaging using a Leica DM IL Led Inverted Microscope with a Phase Contrast System and Fluorescence (Leica Microsystems GmbH, Wetzlar, Germany).

#### 2.3.5. Cryogel Subcutaneous Implantation in Rat Model

Female Wistar rats (17 weeks old; 3 animals, 250 ± 10 g) were housed in temperature- and humidity-controlled (±22 °C, ~52%) individually ventilated cages (12 h light/dark cycle) with free access to water and standard pellet food. All the animal protocols in this study were conducted with the approval of the Ethical Committee of the Grigore T. Popa University of Medicine and Pharmacy of Iasi (no. 17/25 October 2020).

For subcutaneous implantation tests on the dorsum subcutaneous skin, the rats were anesthetized by inhalation with isoflurane gas (Isoflutek 1000 mg/g, Laboratorios Karizoo, Barcelona, Spain) combined with air (5% for induction and 2% for maintenance). The rat back hairs were shaved with the underlying skin cleaned and disinfected with betadine 10% solution. The instruments used in the surgical procedures were sterilized using a glass bead sterilizer (Biobase, Jinan, China). To implant the hydrogels, two parallel 10 mm incisions were cut on the dorsal section of each rat to create small pockets for the biomaterial implantation. One cryogel scaffold was implanted in each subcutaneous pouch, on the left side the bioactive compound (PVA_72000__PEBSA_25/75__Thy_α-Tcp) and on the right side the control one (PVA_72000__PEBSA_25/75_). The incisions were then sutured using D-tek non-absorbable natural multifilament silk 4 (Demophorius Limited, Limassol, Cyprus) and Terramycin spray (Zoetis, Zaventem, Belgium) was topically applied to the surgery sites to prevent infection. All animals were then carefully monitored during the following days for any side effects. Photographs of the scaffold-embedded capsules were taken using a digital camera (Canon, model no. EOS 800D) at 7, 14, and 21 days after materials implantation. At each time point, the rats were euthanized by hyperdose anesthesia and the dorsal skin was carefully resected. The skin sections containing both materials were measured using an electronic caliper, photographed, cut, and fixed in 10% neutral formalin for histopathological examinations.

#### 2.3.6. In Vivo Evaluation of Wound Healing

Adult female Wistar rats (20 weeks old; 5 animals, 280 ± 20 g) were housed in temperature- and humidity-controlled individually ventilated cages (12:12-h light-dark cycle) with free access to water and a standard pellet diet. The animal experiments were performed according to international guidelines and were approved by the Ethical Committee of the Grigore T. Popa University of Medicine and Pharmacy of Iasi, Romania (no. 17/25 October 2020).

The rats were anesthetized by inhalation 5% induction and 2% maintenance isoflurane gas mixed with normal air. The backs of the rats were shaved and the skin was cleaned with 10% iodine povidone solution. Three circular full-thickness cutaneous wounds with a diameter of 15.85 mm were marked and were then carefully excised using sterile sharp scissors. On the first and second wound (counted from the head) the bioactive compound (BC, PVA_72000__PEBSA_25/75__Thy_α-Tcp) and positive control (C+, PVA_72000__PEBSA_25/75_) were placed, and the last excision was left empty, which was considered the negative control (C−). To preserve the dressings, sterile gauze was sutured over the scaffolds using D-tek non-absorbable natural multifilament silk 4 (Demophorius Limited, Limassol, Cyprus).

The day of wound creation was defined as day 0 of the study. For the animal’s pain management, an intraperitoneal injection of 40 mg/kg tramadol was performed on the day of surgery (day 0). Photographs of the wounds were taken using a digital camera (Canon, model no. EOS 800D) at days 0, 5, 10, 15, and 20 during the healing process. The wound sizes were measured on the 5th, 10th, 15th, and 20th day using an electronic caliper. The wound-healing ratio was calculated using Equation (4):

(4) $$\mathrm{Wound}-\mathrm{healing}\;\mathrm{ratio},\;\%=\frac{S_{0}-S_{n}}{S_{0}}\times 100$$

where S_0_ and S_n_ are the wound areas at day 0 and the predetermined experimental day, respectively.

On day 20, the animals were euthanized by hyperdose anesthesia and each full-thickness wound tissue with a minimum of 0.5 cm normal tissue margins was collected for histological studies.

#### 2.3.7. Interleukin-8 Determination by Enzyme-Linked Immunosorbent Assay (ELISA)

Peripheral blood samples were collected to measure the serum level of interleukin (IL)-8 with a commercially available ELISA kit (code ABIN6968034, Antibodies-online GmbH, Aachen, Germany) according to the manufacturer’s recommendation. Briefly, 100 µL of standards, blank, and samples (diluted 1:1 with sample dilution buffer) were added into appropriate wells and incubated at 37 °C for 90 min. The plate was then manually washed 2 times, followed by 100 µL of biotin-labeled antibody addition and incubation at 37 °C for 60 min. The assay proceeded with the addition of 100 µL of HRP-streptavidin conjugate, incubated at 37 °C for 30 min, followed by 90 µL of TMB substrate. After incubation at 37 °C for 30 min, 50 µL of stop solution was added to each well, and the optical density was determined at 450 nm using the Tecan Sunrise Plate Reader, Switzerland. The data were generated in electronic format with the help of the Magellan v 7.2 software. All measurements were performed in triplicate. Afterward, the results were determined by employing the standard curve generated through MyAssays online v R10.2 software [20], where the average absorbance of standards was plotted against their known concentrations (7.813–1000 pg/mL).

## 3. Results and Discussion

### 3.1. Atomic Force Microscopy (AFM)

**Figure 1:**
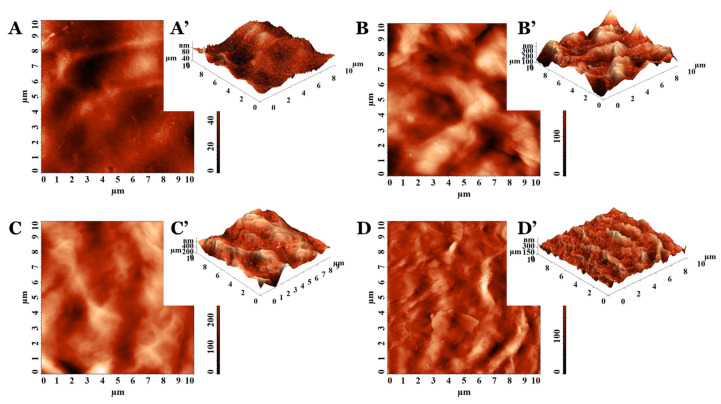
The 2D and 3D AFM images collected on (**A**,**A’**) PVA_72000_, (**B**,**B’**) PVA_72000__PEBSA_25/75_, (**C**,**C’**) PVA_72000__PEBSA_50/50_, and (**D**,**D’**) PVA_72000__PEBSA_75/25_.

**Figure 2:**
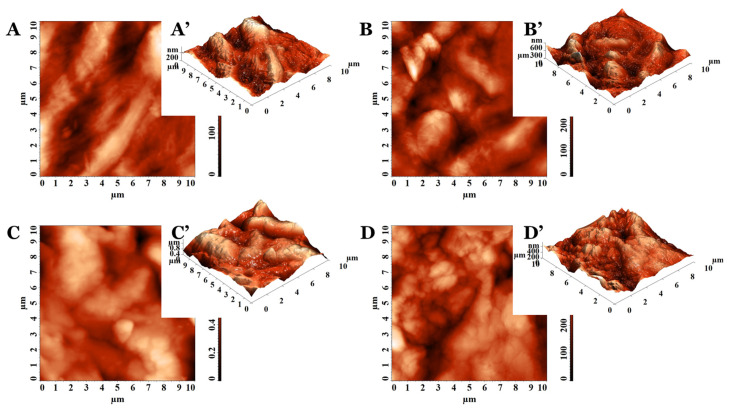
The 2D and 3D AFM images collected on (**A**,**A’**) PVA_145000_, (**B**,**B’**) PVA_145000__PEBSA_25/75_, (**C**,**C’**) PVA_145000__PEBSA_50/50_, and (**D**,**D’**) PVA_145000__PEBSA_75/25_.

The representative 2D and 3D AFM images of PVA_PEBSA cryogel samples are illustrated in Figure 1 and Figure 2. The AFM texture parameters are presented in Table 2. Analyzing the 10 × 10 µm^2^ topographical data characteristics of the cryogels based on PVA (M_w_ = 72,000 g/mol in Figure 1 and M_w_ = 145,000 g/mol in Figure 2), it can be noticed that the surface morphology is influenced by the sample composition. The control PVA_72000_ sample surface (Figure 1A,A’) had a lower root mean square roughness (Sq) (Table 2) than the pristine PVA_145000_ surface (Figure 2A,A’). This indicates that the protuberances become rougher with the PVA molecular weight increase. This aspect was demonstrated on other classes of polymers, namely ZEP520/ZEP7000 (copolymers of α-chloromethacrylate and α-methylstyrene) [21] and polystyrene [22].

The Sq parameter of the PVA_PEBSA systems was influenced by the comonomer EB/SA amount included in the PEBSA composition (Table 2). The same trend was observed for pristine copolymers PEBSA_25/75_, PEBSA_50/50_, and PEBSA_75/25_, respectively [23], with the highest values obtained when using PEBSA_50/50_ (see Figure 1B–D and Figure 2B–D). Regarding the aspect of the surface features, the systems containing PVA_72000_ present sharper morphology (specifically as observed from the 3D AFM images from Figure 1A’–C’) and lower roughness, while the ones with PVA_145000_ have a bumpy morphology, are rounded on the edges (Figure 2A’–C’), and have higher roughness (Table 2). Thus, the rugosity is influenced by the strength of the interactions between the two polymers included in the studied systems structure, depending on the molecular weight of the PVA, but also on the PEBSA composition.

Taking into consideration the values from Table 2 obtained for the morphology entropy (Sent), an indicator of the form–dimension complexity [24], the same trend of variation was observed. The low values calculated for the pristine PVA samples, compared to the corresponding PVA_PEBSA systems, denote distinct degrees of order in the morphological structure surfaces. Nevertheless, by increasing the PVA molecular weight, the organization level decreases: Sent (PVA_72000_) < Sent (PVA_145000_). From the entropy point of view, PVA_PEBSA systems present a morphological disorganization, also intensified with the enhancement of the PVA molecular weight. For each category of PVA_PEBSA system, the highest disorder in morphology was observed when PEBSA_50/50_ was used.

In order to confirm the suppositions offered by the estimation of the morphological entropy, another 3D parameter was calculated, namely the texture direction index of the surface (Stdi). Stdi describes the texture of the relief and the balance between the isotropic character (when it is greater than 0.500 and closer to 1) and the anisotropic one (when its value is less than 0.500 and is as close as possible to 0) [25]. As expected, the results indicated morphologies with predominant orientation (anisotropy) for PVA_72000_ (Stdi = 0.277) and PVA_145000_ (Stdi = 0.291). Instead, all other surfaces of the cryogel samples were isotropic from the topographical perspective, with Stdi being higher than 0.500 (Table 2). In these cases, there is a slight disturbance in the order, induced by the formation of intermolecular bonds as a result of the interactions between the blend partners in the isotropic structure of the cryogel.

**Figure 3:**
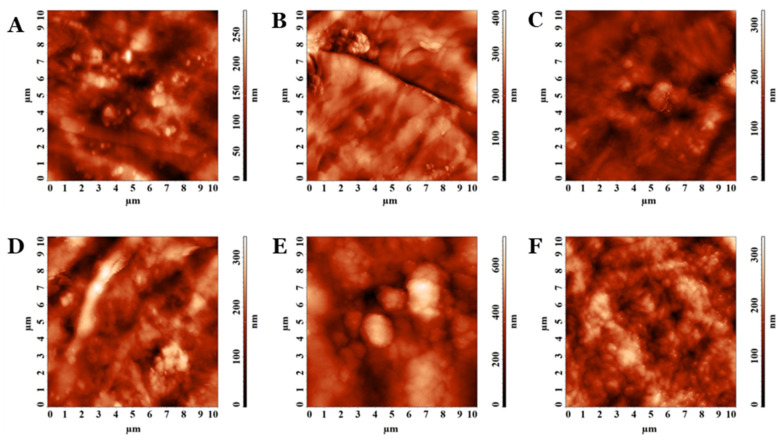
The 2D AFM images collected on (**A**) PVA_72000__PEBSA_25/75__Thy-αTcp, (**B**), PVA_72000__PEBSA_50/50__Thy-αTcp, (**C**) PVA_72000__PEBSA_75/25__Thy-αTcp_,_ (**D**) PVA_145000__PEBSA_25/75__Thy-αTcp_,_ (**E**) PVA_145000__PEBSA_50/50__Thy-αTcp, and (**F**) PVA_145000__PEBSA_75/25__Thy-αTcp.

PVA/PEBSA_Thy_α-Tcp bioactive compounds were also investigated from the morphological point of view using AFM, the height images being presented in Figure 3. The systems with bioactive complexes showed reduced roughness compared to the calculated values for the corresponding matrices (Table 2). In these cases, the molecular weight of the PVA and PEBSA compositions influenced the amplitude parameters. The bioactive compounds having in their compositions as PVA_145000_ showed higher roughness (Sq) and disorder in morphology (Sent) than those with PVA_72000_. Nevertheless, for PEBSA_50/50_, maximum roughness and morphology entropy were obtained for each set, compared to the other compositions of PEBSA_25/75_ and PEBSA_75/25_. The systems keep their isotropic character even after the inclusion of Thy and α-Tcp, and Stdi has values above 0.500, according to Table 2.

From the AFM investigation results, the PVA_PEBSA systems are suitable for tissue engineering scaffolds with an adequate surface roughness of polymeric gels. More than that, the absence of directional alignment, namely the isotropic morphology of the PVA_PEBSA polymer systems, is often preferred in applications where lack of preferential orientation is essential, such as in tissue engineering scaffolds or drug delivery systems.

### 3.2. Water Contact Angle (WCA)

**Figure 4:**
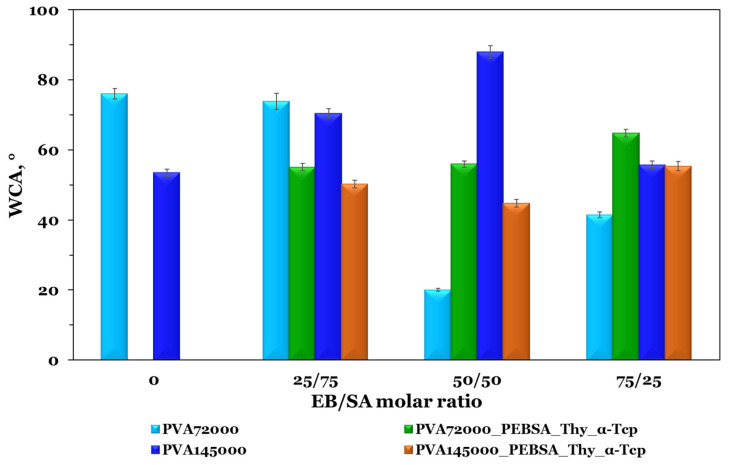
WCA measurements of PVA and PEBSA copolymacrolactone blend films with and without Thy_α-Tcp. Graphical data are expressed as mean ± standard deviation.

The evaluation of the materials wettability, designed for skin wound care, provides information about the hydrophilicity/hydrophobicity of the surface. In order to hold an adequate hydrophilic capacity to absorb wound exudate, the WCA of an ideal wound dressing surface should be smaller than 90° [26]. As shown in Figure 4, the PVA-based films present a low WCA value, with a strong hydrophilic character in the case of the PVA with the highest molecular weight (53.5°). This feature can be attributed to a larger number of hydroxyl groups on the macromolecular chain.

The wettability variation of PVA_PEBSA blends is different and depends on the PVA molecular weight. As already pointed out in a previous study [27], EB governs the hydrophobic character of the PEBSA copolymer. For the PVA_PEBSA samples, the WCA does not vary proportionally with the PEBSA ratio, which indicates that the wettability is highly influenced by the supramolecular arrangement of the polymeric constituents in the blends. The PVA_145000__PEBSA_25/75_ sample has the largest CA of ~88°, placing the sample at the upper limit of the hydrophilic domain (WCA of 90°), suggesting a slight hydrophobic character, whilst the PVA_72000__PEBSA_50/50_ presents a lower contact angle of 20.1°, attributed to a strong hydrophilic surface (see Figure 4). The samples containing PVA with low molecular weight, show a much wider variation in the wettability, while in the case of PVA_145000_-based systems, the variation is narrow. The PVA_145000__PEBSA_75/25_ sample has the lowest contact angle value, while the PVA_145000__PEBSA_50/50_ system registers the highest score, at the upper limit of the hydrophilic domain near the hydrophobic one. This suggests that the PVA low molecular weight presents a wider range of possible interactions with PEBSA.

The hydrophobic/hydrophilic character of the PVA_PEBSA system influences the incorporation efficiency of Thy and α-Tcp, both hydrophobic compounds, but with slightly pronounced lipophilic character for α-Tcp. The addition of Thy and α-Tcp into the PVA_72000__PEBSA-based matrix determines a decrease in WCA for the cryogel containing PEBSA_25/75_, while an increase in WCA is observed for PEBSA_50/50_ and PEBSA_75/25_ samples. In the case of PVA_145000__PEBSA systems, the encapsulation of the bioactive compounds determines a decrease in of WCA for the PVA_145000__PEBSA_25/75__Thy_α-Tcp and PVA_145000__PEBSA_50/50__Thy_α-Tcp samples and an increase for the PVA_145000__PEBSA_75/25__Thy_α-Tcp probe. It can be noted that the WCA differences among all cryogel compositions are small (around 20°) after adding the bioactive compounds, Thy and α-Tcp. The WCA values are in the range of 44° (PVA_72000__PEBSA_50/50__Thy_α-Tcp) to 64° (PVA_145000__PEBSA_75/25__Thy_α-Tcp), thus indicating moderate hydrophilic character surfaces. The enhanced hydrophilic properties of the films through incorporating hydrophobic compounds into theoretically hydrophilic matrices, appears initially unexpected. This can be explained by the supramolecular arrangement of the hydrophobic moieties placed in the inner part of the structures and the hydrophilic ones oriented towards the air interface. Similar results were reported by Stoleru et al. [28] study.

In summary, the WCA is a critical parameter directly related with the compounds ability to interact with hydrophobic or hydrophilic substances. Considering the investigated systems it is clear that the surface-changing ability is dependent on the ratios’ variation between the polymers and the integration of the active compounds.

### 3.3. In Vitro Interaction with Simulated Body Fluids

**Figure 5:**
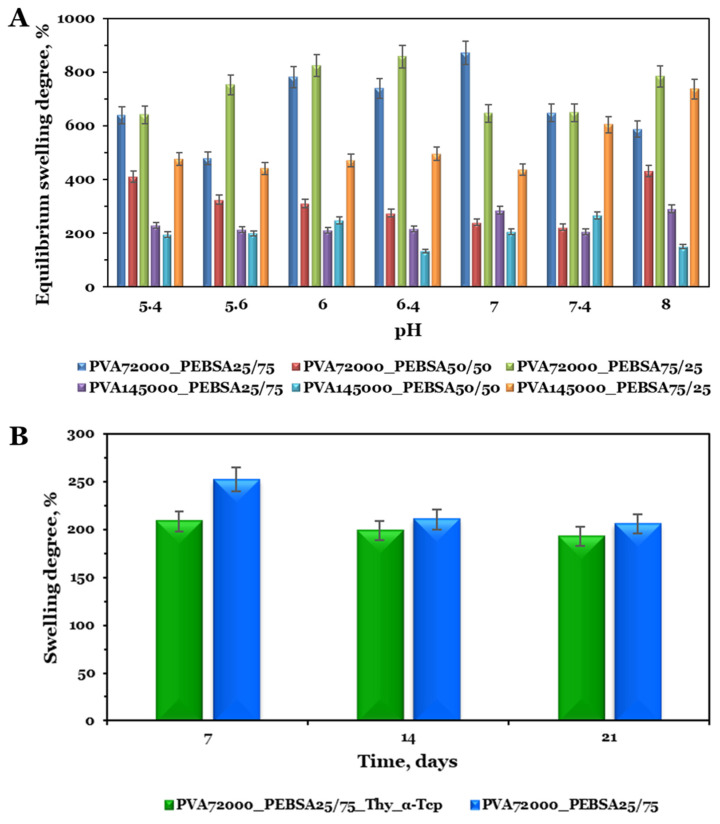
(**A**) Equilibrium swelling degree in PBS solution at different pH values and (**B**) swelling degree vs. immersion time (days) in SBF solution. Graphical data are expressed as mean ± standard error of the mean.

Hydrogel dressings require an adequate fluid assimilation capacity in order to absorb excessive exudates, thus reducing the risk of infection as well as providing a moist local environment [29]. To evaluate the response of the cryogels based on PVA and PEBSA copolymacrolactone in phosphate buffers in relation with the environmental pH variation, the samples were immersed in PBS solutions at 37 °C at different pH values (5.4, 5.6, 6, 6.4, 7, 7.4, and 8, 0.01 M) (Figure 5A). Taking into account the pH fluctuations as the wound progresses toward healing [30], a range of 5.4–8.0 was selected for the swelling studies. Moreover, in order to anticipate the in vivo bioactivity of the cryogel scaffolds, the swelling behavior study of the PVA_72000__PEBSA_25/75__Thy_α-Tcp and PVA_72000__PEBSA_25/75_ systems was performed in SBF solution, a fluid that mimics the inorganic composition of blood plasma [31] (Figure 5B). 

The variation of PVA_PEBSA swelling degree after 48 h, when equilibrium was reached, is depicted in Figure 5A. It is important to mention that all cryogel options displayed maximum swelling degree properties ranging from 200% to 870% in PBS solution. This behavior can be explained both by the hydrophilic/hydrophobic character of the systems attributed to the functional groups of the polymers and by the porous structure of the scaffolds. Analyzing the swelling degree variation at equilibrium with the pH of the samples, it was observed a similar tendency in an acidic and basic pH, with a significant swelling at nearly neutral conditions. Since the SA carbonyl groups are not significantly sensitive to pH [32], an increase in the swelling capacity can be attributed to the formation of additional hydrogen bonds between water and the PVA hydroxyl groups on the macromolecular chains. On the other hand, a high swelling degree is mainly associated with a porous structure. In our previous study [17], the SEM micrographs of the PVA_PEBSA systems showed a high porosity and the presence of PEBSA determined the formation of the network with larger meshes and implicitly the increase in the swelling degree. The sample PVA_PEBSA_50/50_ is the exception due to the ordered and compact structure, which has more intermolecular bonds between the blend partners that limit solvent access to the network meshes (as shown in Figure 5A).

In the second part of the in vitro study, the selected cryogels were immersed in SBF solution for 21 days to obtain data regarding their behavior after subcutaneous implantation in rat model. The variation in the swelling degree over time of PVA_72000__PEBSA_25/75__Thy_α-Tcp and PVA_72000__PEBSA_25/75_ scaffolds are presented in Figure 5B. As expected, the presence of ions (positive—Na^+^, K^+^, Ca^2+,^ Mg^2+^, and negative—HPO_4_^2−^, Cl^−^, HCO_3_^2−^, SO_4_^2−^) [19] in the SBF solution reduces the swelling capacity. For both systems, a higher swelling degree was observed in the first week, nearly 200% corresponding to the PVA_72000__PEBSA_25/75__Thy_α-Tcp system, and 250% for the PVA_72000__PEBSA_25/75_ system, respectively, and a decrease after this period. As the fluid molecules penetrate the immersed polymeric network, which determines the pores expantion, the osmotic pressure decreases [33]. At last, they balance each other and the temporary junctions are dissociated, the degradation of the material starts, and in the case of the PVA_72000__PEBSA_25/75__Thy_α-Tcp system, the release of bioactive molecules starts.

### 3.4. In Vitro Cytocompatibility

**Figure 6:**
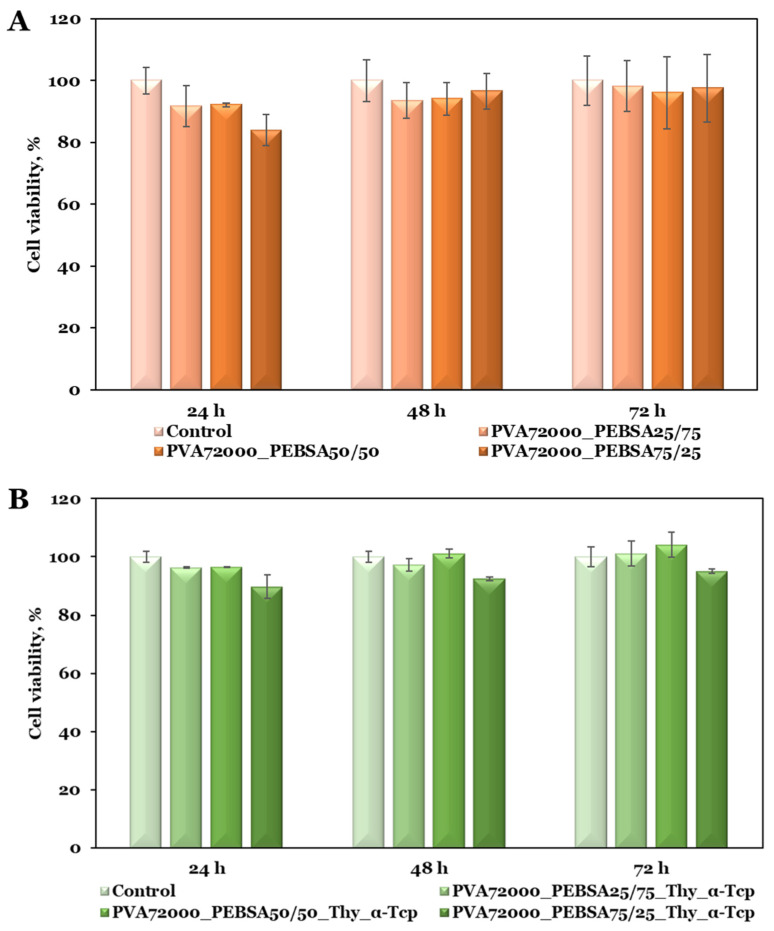
Cell viability data from the MTT assays of the (**A**) PVA_72000__PEBSA and (**B**) PVA_72000_/PEBSA_Thy_α-Tcp systems on BALB/3T3 fibroblasts. Graphical data are expressed as mean ± standard deviation.

Biocompatibility is the fundamental necessity for a material intended to be used in direct contact with skin tissue [34]. Cell viability represents the initial assessment to analyze cellular mitochondrial function upon interaction with new materials [35]. In this regard, the MTT assay was used to evaluate the cytotoxicity of the PVA_72000__PEBSA and PVA_72000_/PEBSA_Thy_α-Tcp systems on BALB/3T3 fibroblasts according to international standards (ISO 10993-5: 2009) [36]. The MTT results (Figure 6A) proved the cytocompatibility of the PVA_72000__PEBSA scaffolds with nearly 90% cell viability after 72 h of incubation. The sample containing the highest amount of SA comonomer (PVA_72000__PEBSA_25/75_) exhibited the most notable results as cell viability increased over time, from 92% at 24 h to 98% at 72 h compared to the control group. As observed from Figure 6B, the interaction of PVA/PEBSA_Thy_α-Tcp bioactive compounds with BALB/3T3 fibroblasts resulted in the same non-cytotoxic character, with the viability percentage exceeding 100% after 72 h of incubation. These results suggest that the incorporation of the bioactive molecular agents into the PVA/PEBSA matrix favored the preservation of cell viability.

**Figure 7:**
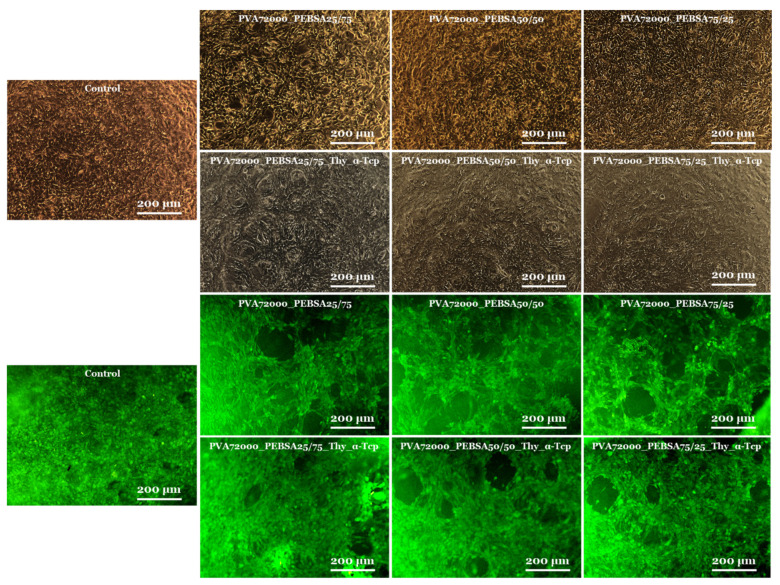
Viable cells and fixed cells, respectively, after 72 h of cell culture with the PVA_72000__PEBSA and PVA_72000_/PEBSA_Thy_α-Tcp systems (10×).

The microscopic examination of the cell culture status after 72 h (Figure 7) sustained the MTT assay data and showed that the cells had a morphology of typical fibroblasts with a homogeneous monolayer comparable in density to the control group [37]. Fluorescence microscope images from live/dead experiments indicated that the majority of fibroblasts were still alive after 72 h. BALB/3T3 fibroblasts in the sample groups showed similar fluorescence intensities compared with the control group [38], and cells reached a confluent monolayer, particularly remarkable in the PVA_72000__PEBSA_25/75_ and PVA_72000__PEBSA_25/75__Thy_α-Tcp systems. Additionally, the BALB/3T3 fibroblasts [14] maintained their normal morphology (as shown in Figure 7) and displayed rapid multiplication in cell culture. The fact that the cells were able to multiply during the experiment indicates that the scaffolds are suitable for cell proliferation.

### 3.5. Cryogel Subcutaneous Implantation in Rat Model

**Figure 8:**
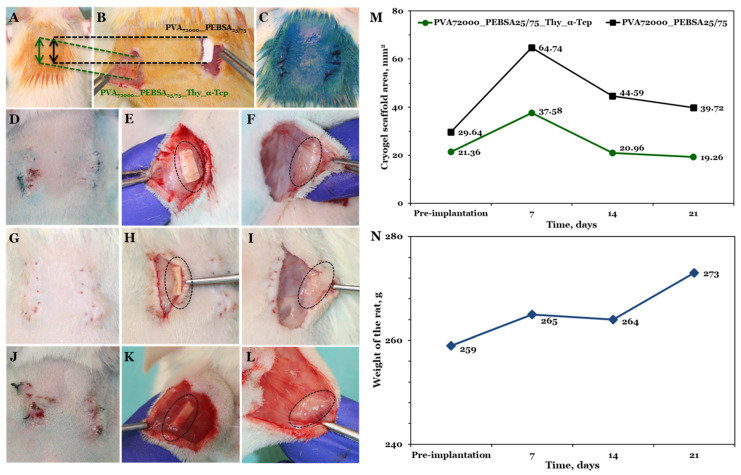
The procedure of cryogel subcutaneous implantation: (**A**) Wistar rat skin prepared for incisions; (**B**) cryogel systems placed subcutaneously; (**C**) sutured and aseptic incisions; capsule formed subcutaneously after (**D**–**F**) 7 days, (**G**–**I**) 14 days, and (**J**–**L**) 21 days; (**M**) evaluation of cryogel scaffold area changes, from implantation up to 3 weeks; (**N**) rat weight changes from pre-implantation to 21 days post-implantation.

During the experiment, the survival behavior, food and water consumption, fur, mucous membranes, and inflammatory processes of female rats were analyzed. The tested cryogel systems did not cause the death of animals, there were no cases of rats exhibiting any pain behavior (assessed by Grimace scale and weight measurements presented in Figure 8N) possible after the materials implantation, and none of the rats displayed visible inflammation or infection.

Both cryogels containing pouches were resected at 7, 14, and 21 days after their implantation and were measured in order to observe their behavior (Figure 8D–L). At all time points, the materials were surrounded by healthy tissue and blood vessels that were proximal or in direct contact, more pronounced in the case of the bioactive compound (PVA_72000__PEBSA_25/75__Thy_α-Tcp). The implanted materials retained their rectangle shape even after the swelling. The pre-implantation bioactive compound (left side) had an area of 21.36 ± 0.66 mm^2^ (Figure 8M) and it was observed to increase as a function of the implantation time in the first week post-implantation, based on the scaffold area that is visible to the naked eye on the skin. On the 7th day post-implantation, the scaffold dimensions increased by approximately 16.22 mm^2^, demonstrating an almost 57% change throughout this time period. Fourteen days post-implantation, the scaffold volume decreases to less than the original dimensions (nearly 98% from the initial area), probably due to the degradation process that occurs in the living organism. Following the implantation, the area of the bioactive compound continued to decline after twenty-one days to a change of nearly 90% compared to the original volume.

In the case of the control cryogel (right side), the initial area pre-implantation was 29.64 ± 2.46 mm^2^, slightly larger than the bioactive compound. After seven days of implantation, the volume of the cryogel increased and the swollen scaffold occupied a double area of about 64.74 mm^2^. After fourteen days, as expected based on the results obtained for the bioactive compound, the PVA_72000__PEBSA_25/75_ cryogel area decreased to about 44.59 mm^2^, and about 39.72 mm^2^ at twenty-one days post-implantation (Figure 8M). This result is directly correlated with the variation in the degree of swelling in the SBF solution where, as concluded above, the degradation behavior occurs and implicitly the release of bioactive molecules begins.

### 3.6. In Vivo Evaluation of Wound Healing

The obtained freeze–thawed scaffolds were evaluated for the in vivo wound healing assay in the full-thickness wound excision rat model. For this purpose, the PVA_72000__PEBSA_25/75__Thy_α-Tcp bioactive compound with superior antimicrobial activity [16] was selected as the experimental group, and the PVA_72000__PEBSA_25/75_ system was selected as the positive control.

**Figure 9:**
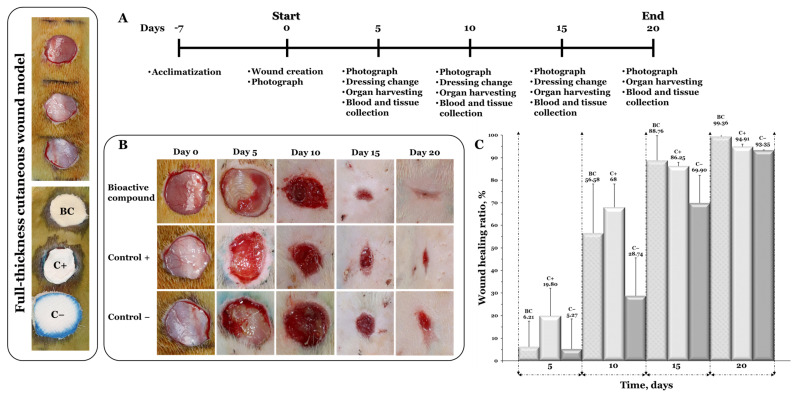
(**A**) Experimental design of the in vivo wound healing assay; (**B**) the images of in vivo wound closure studies for bioactive compound (BC), positive control (C−), and negative control (C−); (**C**) the wound healing ratio at days 5, 10, 15, and 20 for wound treated with bioactive compound (BC) and positive control (C−) in comparison to the negative control (C−). Graphical data are expressed as mean ± standard deviation.

The percent wound healing ratio was calculated at specific time points (on the 5th, 10th, 15th, and 20th day) in this 20-day study. On the 5th day, the healing process was observed in both systems-treated animals as compared with the negative control, whereas maximum wound size reduction was observed in rats treated with PVA_72000__PEBSA_25/75_ (19.80%). A similar trend for the wound healing ratio was witnessed on the following days and on the 10th day an up to 68% healing ratio was recorded in the bioactive compound and PVA_72000__PEBSA_25/75_-system-treated groups as shown in Figure 9. After fifteen days of healing, the wound closure of the bioactive-compound-treated group was 88.76%, which was about 2.5% higher than that of the control system and 18.86% higher than that of sterile gauze (negative control). The study was continued, and on the 20th day, almost complete wound healing was observed (99.36 and 94.91%) in groups treated with both systems.

### 3.7. Inflammatory Cytokine Levels

**Figure 10:**
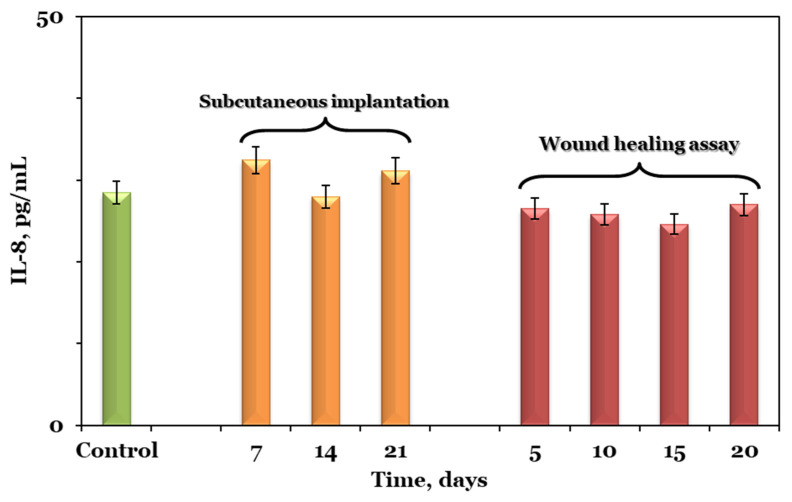
The serum inflammatory cytokine levels in subcutaneous implantation and wound healing assay at different time points in comparison to control. Graphical data are expressed as mean ± standard error of the mean.

The serum inflammatory cytokine levels in both studies, namely the subcutaneous implantation and wound healing assay, at different time points, are shown in Figure 10. Cytokines are low-molecular-weight glycoproteins with an important role in many biological events, such as cellular growth, inflammation, immunity, and tissue repair. As a research hot spot, IL-8 plays a role in the immigration of neutrophils to the area of inflammation, frequently evaluated for the early detection of inflammatory lesions in the areas that are in contact with scaffold components [39].

The content of IL-8 in the control group was 28.45 ± 2.55 pg/mL, while in the experimental group from the subcutaneous implantation model it was 32.34 ± 0.33, 27.96 ± 4.23, and 31.12 ± 1.58 pg/mL at seven, fourteen, and twenty-one days post-operation, respectively. The same trend was also observed for the experimental group in the wound healing assay where the content of IL-8 at five, ten, fifteen, and twenty days post-surgery was 26.51 ± 4.32, 25.79 ± 2.31, 24.59 ± 2.75, and 27 ± 04.77 pg/mL, respectively. Compared with the value in the control group, the IL-8 content in both experimental groups showed no significant change at any of the studied time points.

## 4. Conclusions

Considering the ongoing developments and the constant insertion of innovative wound dressings, it is clear that the wound-dressing market offers plentiful opportunities to address the diverse needs of patients. Our study describes the preparation, characterization, in vitro and in vivo evaluation of the PVA/PEBSA platform embedded with Thy and α-Tcp, for skin wounds healing. The topographical data characteristics point out that the surface morphology is influenced by the sample composition—namely the molecular weight of PVA and the ratio between the EB and SA comonomers of the copolymacrolactone system, and also, PVA_PEBSA systems are suitable for tissue engineering scaffolds with an adequate surface roughness. Water contact angle measurements confirmed the important role of system composition as well in obtaining cryogel structures with desired properties, and the ability of the investigated systems to adjust their contact angle based on the active component incorporation. These data are well corroborated with the in vitro interaction with simulated body fluids, behavior highly influenced by the supramolecular arrangement of the polymeric constituents in the blends. The in vitro cell viability assay results displayed good cytocompatibility with the percent of viable cells exceeding 90% after 72 h of incubation for BALB/3T3 fibroblasts. Animal testing confirmed their biocompatibility, integration with host tissue, and the absence of inflammatory processes. The bioactive compound significantly enhanced the healing process of full-thickness excision wounds in a rat model. Given the encouraging results, PVA/PEBSA cryogels with dual activity can be considered promising wound dressings. Nevertheless, the bioactive compound high potential should be evaluated on in vivo infection models, monitoring the remission of inflammation and wound healing period. These additional tests would illustrate the applicability of the systems in clinical antimicrobial and wound healing therapy. It will take more time to assess the safety [40] of clinical PVA/PEBSA_Thy_α-Tcp applications.

## Figures and Tables

**Table 1:** Samples’ name and bioactive compound preparation.

Sample	PVA:PEBSA Ratio	Composition for a Sample Volume of 5 mL			
		PVA (g)	PEBSA (g)	Thy (g)	α-Tcp (g)
PVA_72000__PEBSA_25/75_	2:1	0.132	0.066	–	–
PVA_72000__PEBSA_50/50_	2:1	0.132	0.066	–	–
PVA_72000__PEBSA_75/25_	2:1	0.132	0.066	–	–
PVA_145000__PEBSA_25/75_	2:1	0.132	0.066	–	–
PVA_145000__PEBSA_50/50_	2:1	0.132	0.066	–	–
PVA_145000__PEBSA_75/25_	2:1	0.132	0.066	–	–
PVA_72000__PEBSA_25/75__Thy_α-Tcp	2:1	0.132	0.066	0.066	0.066
PVA_72000__PEBSA_50/50__Thy_α-Tcp	2:1	0.132	0.066	0.066	0.066
PVA_72000__PEBSA_75/25__Thy_α-Tcp	2:1	0.132	0.066	0.066	0.066

**Table 2:** AFM texture parameters collected on the scanned surfaces of 10 × 10 µm^2^.

Sample	Sq (nm)	Sent	Stdi
PVA_72000_	15.1	10.788	0.277
PVA_72000__PEBSA_25/75_	62.6	12.743	0.716
PVA_72000__PEBSA_50/50_	68.5	12.847	0.556
PVA_72000__PEBSA_75/25_	41.9	12.183	0.529
PVA_72000__PEBSA_25/75__Thy_α-Tcp	37.7	12.052	0.756
PVA_72000__PEBSA_50/50__Thy_α-Tcp	46.7	12.324	0.578
PVA_72000__PEBSA_75/25__Thy_α-Tcp	31.3	12.272	0.643
PVA_145000_	57.9	12.620	0.291
PVA_145000__PEBSA_25/75_	82.5	13.091	0.530
PVA_145000__PEBSA_50/50_	158.8	13.886	0.576
PVA_145000__PEBSA_75/25_	80.7	13.030	0.549
PVA_145000__PEBSA_25/75__Thy_α-Tcp	47.5	13.263	0.575
PVA_145000__PEBSA_50/50__Thy_α-Tcp	96.73	13.279	0.627
PVA_145000__PEBSA_75/25__Thy_α-Tcp	43.55	13.266	0.555

## Data Availability

Data are contained within the article.
